# Families' Strategies for Navigating Care for Their Child With Cerebral Palsy: A Qualitative Study

**DOI:** 10.1111/hex.70197

**Published:** 2025-03-15

**Authors:** Silje Askeland, Veslemøy Guise, Karina Aase, Maren Kristine Raknes Sogstad

**Affiliations:** ^1^ SHARE – Centre for Resilience in Healthcare, Faculty of Health Sciences, University of Stavanger Stavanger Norway; ^2^ Centre for Care Research, Department of Health Science in Gjøvik NTNU Gjøvik Norway

**Keywords:** cerebral palsy, families' care needs, families' experiences, families' strategies

## Abstract

**Introduction:**

Families of children with medical complexities, like cerebral palsy (CP), often interact with multiple service providers across healthcare, education, social services, and family support sectors. To navigate these services, families shoulder various responsibilities, such as managing appointments, understanding different service systems, and advocating for their child's needs. However, our understanding of how families navigate these services remains limited. Therefore, this study explores families' strategies for navigating services for their child with cerebral palsy.

**Methods:**

Data were gathered through interviews with six families who each have a child diagnosed with CP aged between 8 and 12 years old. These interviews involved both children and parents and were conducted in three consecutive semi‐structured sessions with each family. Additionally, observations were conducted during multidisciplinary coordination meetings held at the children's schools, involving parents and service providers.

**Results:**

To navigate services, parents applied strategies to (1) become experts on both their child's diagnosis, challenges, care needs and on the services available; (2) act as proactive participants in their child's care; and (3) manage day‐to‐day care. In doing so, families contributed to the provision of family‐centred services according to their care needs.

**Conclusion:**

Families make use of several different strategies to navigate the services. By applying these strategies, they effectively express their care needs and facilitate tailored services, thus contributing towards a family‐centred approach. This highlights the importance of supporting the strategies used by families when collaborating with the services.

**Patient or Public Contribution:**

Families actively participated in shaping the study by engaging in a series of interviews, discussing topics important to them, and reviewing the information provided. This approach ensures that their experiences and needs are accurately captured and addressed. Additionally, families shared their thoughts on how services could be improved to better meet their care needs.

## 1 Introduction

Families of children with cerebral palsy (CP) often interact with numerous service providers across the healthcare, education, social services and family support sectors [1, 2]. To ensure high‐quality care, these services need to be offered in an effective, safe and family‐centred manner that actively involves patients and their carers [3, 4]. However, families often find these services fragmented and challenging to navigate, hindering their access to necessary support [5, 6, 7]. Research describes situations where diverse services are provided separately by different providers, impeding the delivery of integrated and holistic care tailored to families' needs [7, 8, 9]. Additionally, families of children with chronic illnesses, such as CP, require better coordination, more information, increased respite care and enhanced therapeutic and peer support [6, 8, 9, 10, 11, 12, 13]. These needs are particularly pronounced for children with both behavioural and physical needs [8], such as when a CP diagnosis is accompanied by additional diagnoses [14, 15].

The burden of care on families often exceeds service providers' assumptions [16, 17]. Research indicates that families in several cases use significant resources to find their way through the services and to obtain the necessary help for their child [18]. Families of children with chronic illnesses and disabilities take on various responsibilities and tasks. To navigate the care needed, parents proactively seek health information and advocate for their child by directly communicating care needs and requesting services [17, 19, 20, 21, 22, 23].

Despite this knowledge of the types of responsibilities these families often have to shoulder, our understanding of families' strategies for navigating multiple services remains limited. Therefore, this study aims to explore families' strategies in navigating services for their child with CP through the following research question:

– How can family strategies used to navigate services for children with CP be described?

## 2 Methods

A qualitative study involving semi‐structured interviews with families and observations during multidisciplinary coordination meetings [24, 25, 26] was conducted to explore families' strategies in navigating services for their child with CP.

### 2.1 Context

Through the Norwegian welfare system, children with CP and their families have access to a comprehensive range of services, including health and care services, labour and social services, educational services and children and family services offered by various municipal, regional and governmental units [27, 28]. These services encompass assessments and treatment within specialist healthcare, which subsequently trigger follow‐up services in primary healthcare [29, 30, 31]. Additionally, following assessments and decisions in specialist and primary healthcare, children receive social and educational assistance in kindergarten and school [32, 33, 34]. Furthermore, the specific challenges and needs of the child and family grant them rights to financial and social support services [35, 36], as well as family counselling services [37, 38]. In a previous study, we described the complex care landscape that families of children with CP in Norway must navigate [39]. To assist families with care coordination, children have the right to an assigned coordinator within the primary healthcare services. This coordinator is responsible for organizing multidisciplinary coordination meetings between the various providers involved in the family's services [40, 41].

### 2.2 Recruitment and Participants

Families were recruited through the Child and Adolescent Habilitation Unit at a university hospital in Norway. Before a scheduled CP consultation, they were informed about the study and invited to participate. Families who met the inclusion criteria of having a child aged 6 to 12 years with a primary diagnosis of CP and who agreed to participate provided their contact information, allowing researcher SA to contact them. This process led to the inclusion of six families with children aged 8 to 12 years old. An overview of the participants is provided in Table 1. Customized information letters were given to both the children and their families, outlining the study, and emphasizing their right to withdraw at any time. During the initial meeting with each family, the information letter was reviewed with parents and children. After parents and children confirmed their participation in the study, parents signed a written consent form on their own and their child's behalf. All families included a mother, a father, and a child.

**Table 1:** Overview of study participants.

Families	Child's age	CP diagnosis	Comorbidity
Family 1	8	Gross motor function classification system (GMFCS) level 1, diagnosed at the age of 4 years	Attention Deficit/Hyperactivity Disorder (ADHD), diagnosed at the age of 7 years Learning difficulties
Family 2	12	GMFCS level 1, diagnosed at the age of 8 years	ADHD, diagnosed at the age of 7 years Autism spectrum diagnosis, diagnosed at the age of 8 years Learning difficulties
Family 3	8	GMFCS level 3, diagnosed at the age of 1 year	Epilepsy, diagnosed at the age of 3 years Learning difficulties
Family 4	9	GMFCS level 4, diagnosed at the neonatal stage	Epilepsy, diagnosed at the age of 1 year Cognitive disability, diagnosed at the age of 8 years
Family 5	9	GMFCS level 3, diagnosed at the neonatal stage	Epilepsy, diagnosed at the age of 2 year Learning difficulties
Family 6	9	GMFCS level 1, diagnosed at the neonatal stage	ADHD, diagnosed at the age of 7 years Learning difficulties

### 2.3 Data Collection

Data were collected through individual interviews with children and their families, and during observations in multidisciplinary coordination meetings conducted at the child's school. Interviews and observations were carried out in parallel.

#### 2.3.1 Interviews With Children and Their Families

Between April 2021 and December 2022, each family participated in a series of three semi‐structured interviews. These interviews focused on the services provided to the child and the support extended to the family. The initial interview served to familiarize the researcher with the family, their experiences with service provision, collaborative efforts, concerns, care requirements, and preferences. Subsequent interviews expanded on previously discussed topics, addressed emerging care needs and delved into matters of significance to the family. Employing a semi‐structured guide, the interviews covered essential themes while allowing flexibility for families to discuss topics of importance to them [42]. Throughout the interviews, the researcher remained responsive to the needs of both parents and children, ensuring that questions, discussions, and interview duration were tailored accordingly. Children who could do so participated in the interviews alongside their parents. Before each session, children were briefed on the value of their input, assured of the conversation's brevity, and given the freedom to end the interview at any point if they wished. Questions to children were tailored to their age, using drawings to illustrate their interactions with the services [43, 44, 45]. During the interviews, the children were encouraged to share recent experiences with the care services, such as someone discussing their health or engaging in exercises or tests with them. After approximately 20–35 min, the child's portion of the interview concluded and continued with the parents. Some children chose to remain with their parents for the rest of the interview, while others chose to leave the interview context. The timing, location, duration, and participants in the interviews were varied and aligned with the families' preferences, logistical constraints, and COVID‐19 protocols. Interview duration ranged from 60 to 120 min, with nine sessions held at families' homes, seven conducted remotely, and two hosted at the researcher's office. Siblings of the children were present at certain interviews, while others involved only one parent.

#### 2.3.2 Observations

Observations were conducted during multidisciplinary coordination meetings featuring four of the families. These meetings took place at the child's school and varied in length between 67 and 83 min. Participants included parents and service providers responsible for the child's follow‐up, such as teachers, assistants and a health nurse from the school, advisors from the pedagogical psychological service and providers from municipal services and specialist healthcare. Although children were invited to participate, they chose not to. To ensure the child's voice was represented, parents were asked about the child's preferences for follow‐up activities as well as their care needs. Additionally, the child's teacher or teaching assistant talked to the child before the meeting. The observations aimed to gather information about the collaboration between service providers and each family. Notes were taken during the observations using an observation guide, which aimed to collect information about topics discussed, perspectives addressed, the role of families, strategies used by families to communicate their care needs, communication from service providers to families and follow‐up activities agreed upon during the meeting. The researcher introduced herself, the study and the purpose of the observations at the beginning of each session. She responded to any questions from the participants but did not take an active role in the meetings [46, 47].

### 2.4 Analysis

To explore the strategies families of children with CP use to navigate services, reflexive thematic analysis was used to identify, analyze and report patterns [48]. This analytical process includes six steps, from transcription of interviews to the final report [49]:

1. Transcription: The researcher transcribed the interviews and reviewed them alongside notes from the observations to establish the initial data material.
2. Coding: Using the programme Nvivo, statements from the interviews and observations were coded to capture families' care needs and navigation strategies.
3. Theme development: The established codes were organized into potential themes, reflecting commonalities and patterns across the data set.
4. Alignment and merging: Themes and sub‐teams were assessed against the coded extracts to ensure alignment with the data. Similar sub‐themes were merged for clarity and coherence.
5. Evaluation and refinement: Each theme was evaluated in the context of the overall analysis narrative to ensure clear definitions and names that accurately represented the data.
6. Synthesizing and reporting: Finally, the findings were synthesized into the final analysis report, presenting a narrative that addressed the research question and provided insights into families' strategies to navigate services.

### 2.5 Ethical Considerations

Children with CP and their families constitute a vulnerable patient group that requires special ethical consideration [40]. Incorporating children's perspectives into research necessitates safeguarding their well‐being and interests, ensuring that the patient group benefits from the knowledge generated, and demonstrating that conducting the research with adult participants would not produce equivalent results [50, 51]. These criteria were fulfilled, and ethical approval was secured from multiple entities, including the Regional Committee for Medical and Health Research Ethics in Norway (ref. no. 117063), the Norwegian Agency for Shared Services in Education and Research (ref. No 661345), the Research Department at the participating university hospital and the Research Departments at the participating municipalities.

## 3 Results

The families' journeys from their initial encounters with services to their current level of support, as well as the strategies used to navigate them, reflected changes in care needs according to their child's age, transitions in care and emerging challenges. The follow‐up services they received varied among the families, influenced by their child's gross motor function level and any additional diagnoses. All families had undergone periods of assessment for additional diagnoses at different stages of their child's development. Families described having their care needs met for specific issues, however, challenges emerged when addressing difficulties affecting the child's functioning across various domains, necessitating services from diverse care providers for such issues as social, cognitive and behavioural problems. In these situations, long waiting times and service unavailability posed obstacles.

Families also experienced periods of discontinuity in service providers and were repeatedly asked by new service providers about previous decisions regarding diagnosis and treatment. Additionally, contextual factors such as having other children with diagnoses, parental illness, parents' divorcee and involvement with family counselling and child protection services impacted these families' overall situation leading to additional care needs. The children also valued continuity in service providers as well as the provision of easily understandable information.

Based on their experiences with service providers and how the services were organized, the families had developed different strategies to navigate the services they needed as displayed in Table 2.

**Table 2:** Family strategies, themes, and sub‐themes.

Themes	Sub‐themes
Become experts	Search for information Establish a supportive network
Act as proactive participants	Create collaborative relationships Communicate care needs and identify services
Manage day‐to‐day care	Coordinate services Facilitate and perform care

The reported themes and sub‐themes were all detailed and expressed in interviews with all the families. Additionally, the information shared in interviews was, on several occasions, observed during the meetings, as demonstrated under the sub‐themes.

### 3.1 Become Experts

The families described a strategic process of becoming an expert on their child's diagnosis, challenges and care needs, as well as on service availability. They actively sought information and established a supportive network.

#### 3.1.1 Search for Information

Throughout their child's development, families encountered different situations arising at various stages where they experienced new challenges and accordingly needed information that they did not receive automatically and often found difficult to obtain:

“You don't really receive information without asking. And it's difficult to know what to ask for during the consultation. Especially when the doctor is running late and there are many topics that need to be addressed in a short period of time. Additionally, the information provided can be difficult to understand, and the questions always arise after a while once you've had the opportunity to think it through.”

(Mother, Family 2)

Typically, these situations occurred during periods of assessment or when new challenges emerged in the child's life. Several parents described feeling unsure about the implications of the information they received during consultations, which led to additional questions. Although children also attended these consultations, the information was often not tailored to their level of understanding. Consequently, parents struggled to answer their child's questions about the topics discussed. Upon returning home, parents often turned to the internet for more information. These searches could be overwhelming and introduce additional worries, so parents were critical of which sources they used. They preferred national official websites and hospital websites for health‐related and diagnosis‐specific information, which were seen as reliable, easier to understand and targeted at parents. Parents appreciated it when these webpages included information targeted at children, which they found useful in preparation for explaining the diagnosis and accompanying challenges to their child. Additionally, parents sought information through interest organizations specific to the diagnosis:

“We have mostly found the information we need through searches on the internet. In that way we have gained a good overview of the various issues accompanying the CP and ADHD diagnoses, what we can do as parents, and what services are available and possible to apply for. I think that we might not receive all this information because the issues [our child] experiences due to the CP diagnosis are quite limited. But it's all those other things, especially the ADHD diagnosis. We feel like [our child] falls between two stools because [our child] is as well as [our child] is, but there is a lot that the eye can't see.”

(Father, Family 1)

Parents described acquiring knowledge as a process that required time. In addition to searching for information after consultations, they sought information before the next consultation to be prepared. To obtain the necessary expertise, parents compared information before discussing it within the family, or with other parents in a similar situation. Parents that had other children, felt more informed due to their parenting experience and were more confident at being able to find useful information. Conversely, first‐time parents emphasized the value of receiving support from staff at the children's health centre to help them understand hospital‐provided information during their child's first years of life.

#### 3.1.2 Establish a Supportive Network

In their interactions with service providers, parents encountered situations where they needed a supportive network to assist them.

To establish such a network, parents connected with each other through rehabilitation programmes for their children. Some families met in weekly exercise groups for children with CP, while others bonded during stays at rehabilitation centres. These connections provided mutual support, shared experiences and strategies for obtaining necessary services. Parents would for example discuss maintaining services during the school year or school transfer considerations to better meet their children's needs. They also exchanged information about available services and exercise programmes which they could apply for. Additionally, parents shared experiences on supporting their child, including motivating for exercise, encouraging the use of aids and providing emotional support. Children who participated in rehabilitation programmes appreciated meeting peers with similar challenges as part of their supportive network:

“It was a lot of fun to be at [the rehabilitation center]. I could try so many different activities that I never had done before. And everyone could participate and do the same things. It was nice that not everyone was better than me at everything.”

(Child, Family 3)

All families joined one or more interest organizations to receive information, connect with other families and participate in social and educational events. Parents found the courses offered by these organizations invaluable for gaining knowledge. Additionally, they provided advice when specific care needs were not met and served as a platform for parents to influence services by offering collective feedback:

“I have become a member of the association's local parental team. In that way, I can contribute to arrangements and support for families that they need and value. And it is an opportunity to influence the services where we emphasize parents' needs through the association's feedback.”

(Mother, Family 2)

### 3.2 Act as Proactive Participants

Families established collaborative relationships with service providers, communicated their care needs, and identified services to act proactively.

#### 3.2.1 Create Collaborative Relationships

Families emphasized collaborative relationships with service providers as important, especially when facing challenges during their child's long‐term care journey. To them, a collaborative relationship meant having providers who were available, committed to follow‐up care, acknowledging of parents' expertise and who showed mutual respect. To further foster collaborative relationships, families adopted an open approach:

“We are completely open when we talk to care providers. If we clearly communicate where we can contribute, where we have competence and resources, and where we need help, it is much easier for everyone. For example, I can provide support and motivation, follow up on exercises and stretching, and facilitate [my child's] day. But it is more challenging now that [my child] is older and lags behind peers socially and at school, when new challenges arise, and [my child] is more aware of the limitations. In that area, I find it difficult to know what to do. You know, if everyone is open about their efforts to follow up on care and where they fall short, it is easier to navigate these challenging situations.”

(Mother, Family 3)

Families found it easier to have their requests addressed when they engaged in positive dialogue and expressed appreciation for the support they received. During their communication with providers, families initially provided positive feedback on the efforts made to follow up on their child's care:

“During the meetings, we always first emphasize the positive things and show that we appreciate the follow‐up care because I think it is much easier to thereafter discuss what we could do better. I think it is smart to start with the positive things to get more goodwill in return.”

(Mother, Family 5)

When families experienced situations where their care needs were not met, they emphasized the importance of distinguishing between the providers and the services. They believed that the providers tried their best to meet the child's and family's care needs but had restrictions they needed to comply with. This distinction was crucial to maintaining a collaborative relationship with service providers when discussing disagreements. Additionally, this approach was described as important when parents needed to formally complain about the services provided or seek others who could provide follow‐up services for their child. Families' efforts to create collaborative relationships were also observed in multidisciplinary meetings, where they engaged in open dialogue with service providers, expressed appreciation for the efforts to provide necessary services, and distinguished between the service providers and the limitations of the services.

#### 3.2.2 Communicate Care Needs and Identify Services

Families described how they first directly communicated their care needs before requesting and suggesting follow‐up approaches. To directly communicate their care needs, parents developed a condensed narrative to present during meetings. This narrative covered their journey with the services throughout their child's development, including diagnoses, challenges, care needs, current status and future concerns. Some of the parents had this narrative in a written form and kept it in a folder along with assessment reports confirming the child's diagnoses. Additionally, parents provided direct feedback on service approaches and requested further follow‐up services. For instance, some families opted to delay the testing of medication for ADHD‐related problems and instead requested additional support for their child while at school. Other families told service providers that they needed a stable period for their child where previously set diagnoses and decisions were not questioned or reconsidered.

To receive necessary help and support, families frequently requested specific services they had learned about, such as exercise groups or rehabilitation stays. Families frequently experienced situations where services were discontinued once their child had achieved set goals. Parents perceived this as lacking a holistic approach, where long‐term planning and the child's potential for future development were not adequately considered. In these situations, parents found themselves needing to reapply for services, where they had to argue the need to set new goals that would contribute to their child's further development:

“You never enter a meeting with relaxed shoulders. You have done your homework and are well prepared because you do not receive anything without fighting for it. You need to be an advocate for your child in all situations because, as a parent, you know your child best, their needs, and what works.”

(Mother, Family 6)

Families repeatedly requested both new and ongoing assessments and subsequent reports from various care units. Despite these efforts, families would often encounter long processing times and a lack of service offerings to address emerging needs. This was usually due to either a lack of continuity in follow‐up personnel or differing perspectives on appropriate treatment decisions between parents and professionals. In such cases, parents sought assistance from other public service providers. Several families described contacting multiple providers in the public sector to request the same information and referrals to hospital departments and diagnosis‐specific units. While some families received the desired referral after multiple requests, others did not obtain the referrals they wanted.

In other instances, parents had to suggest alternative follow‐up approaches or find alternate resources to support their child due to existing services being inadequate or because of personnel shortages. Two of the families described how they had changed their child's school in order for the child to receive the necessary support in class. Families also turned to private providers when the desired services and support were not available through public channels, including treatments and care provided by physiotherapists and chiropractors:

“It is much better to go to the private physiotherapist because then I can exercise once a week all the time, instead of several times a week for just shorter periods. And the physiotherapist I have now won't quit. Before that, [at the municipal services], I had three or four different physiotherapists in one year.”

(Child, Family 2)

During multidisciplinary meetings, families' efforts to communicate their care needs and identify services were observed. In these meetings, families recounted their journey with the services, including diagnoses, challenges, care needs, current status and future concerns. They requested new and ongoing assessments and subsequent reports, asked for specific services and suggested other follow‐up approaches.

### 3.3 Manage Day‐to‐Day Care

To manage day‐to‐day care, families actively coordinated services between all providers involved in the child's care, in addition to facilitating and undertaking care themselves at home.

#### 3.3.1 Coordinate Services

Families described spending considerable time coordinating specialists, therapists, educators and community support services. This coordination was essential to secure the support their child needed:

“As a mother, I have always had the coordinating role in [my child's] care. During periods with several challenges, it has been a lot of work and very time‐consuming. It was eventually decided that someone from the municipal services should have this role, but it fell through. Then [my child's] physiotherapist tried to help us, but that did not work. As a physiotherapist, she does an amazing job with [my child], but the coordination is too much on top of her regular work. So, I need to take on those things myself.”

(Mother, Family 4)

Some families found this coordination work particularly difficult during the child's early years before they had gotten the necessary support services established. It became easier once the system was set up according to the family's needs, as much of the work then involved maintaining collaborations between service providers. However, others described the need for significant time and resources for coordination during periods when new challenges emerged in the child's life, particularly during transitions and when service providers were replaced. Several families found it difficult to relinquish the coordination responsibility:

“We, as parents, need to constantly keep a hold of the thread in the follow‐up services. We need to have the resources to anticipate challenges and care needs before they occur, and to coordinate and share this information among all the participants in [our child's] care. It is difficult to put this coordination responsibility aside and trust that it will be taken care of. Additionally, our experience is that this is what we need to do to get the support we need.”

(Mother, Family 3)

To coordinate services, families obtained and shared information between all involved providers, corrected information during consultations and meetings, and identified interdependencies in their child's care. Families brought with them their folder of important documents, knowing from experience that such information was not automatically shared between providers. They sent regular emails to providers with updates on current issues and further needs such as medication status or the status of support needs at school. Occasionally, families corrected information during consultations if it had not been noted in the child's records, for example, if service providers assumed that referrals or reports had been sent and that specific challenges had been discussed with the family. Additionally, assessment reports and applications for services or equipment often failed to include all relevant information. As a result, families described taking on a quality assurance role, constantly reviewing and distributing information to ensure accurate descriptions of their child's difficulties and care needs.

Families also identified interdependencies in their child's care, particularly when services from different sectors needed to be coordinated in the right order. Examples of such interdependencies were having to plan follow‐up exercises and stretching from physiotherapists located within the municipal healthcare services after receiving Botox injections from providers in the specialist healthcare setting, or ensuring medical or psychological assessments by specialist healthcare providers before educational providers could do their pedagogical assessments. In these situations, parents contacted service providers to inform them of planned consultations and to reschedule appointments to ensure that the correct sequence of follow‐up care was provided. They also contacted service providers to arrange multidisciplinary meetings when these had not been scheduled as agreed. Some parents furthermore recorded their own minutes from these meetings and repeatedly chased agreed follow‐up actions, especially in situations where a service coordinator had not yet been allocated. The coordinating role of parents in their child's care become apparent also during multidisciplinary meetings, where parents share information with participants, including updates on their child's care services. They corrected information and addressed interdependencies during discussions on follow‐up approaches. Additionally, they reported on conversations conducted via phone calls and emails and provided copies of documents such as assessment reports and applications.

#### 3.3.2 Facilitate and Perform Care

Despite the children having different gross motor function levels and thus different associated challenges or additional diagnoses, all families facilitated and performed care tasks to meet their child's needs across a variety of areas. This included organizing daily routines, supporting participation in exercise, rehabilitation groups and leisure activities and arranging care provided by personal assistants in the home. For children with additional diagnoses of ADHD and autism spectrum diagnosis, they carefully prepared them for activities and routines for the day or week ahead. Other families dedicated time to following up on their child's use of aids, ensuring transport to and from school and managing exercise groups. One family explained how they employed personal care assistants through financial support from public services. This work involved organizing the assistants' shift schedules, ensuring that necessary equipment was available, and providing training and support for the assistants:

“It works very well when everything goes smoothly and there are no challenges in getting the shifts covered. However, when illness occurs [among the assistants], we become very vulnerable. It's not always easy because the assistants often work this job part‐time alongside other commitments. So, when I hire assistants, I always look for those who are dedicated and genuinely want to, for example, take [my child] out in the afternoon for a walk to ensure there's meaningful activity in the day.”

(Mother, Family 5)

All families provided care tasks themselves after agreeing on follow‐up plans with service providers. This included managing tube nutrition, and facilitating and participating in general physical activity to improve or maintain the child's functional level:

“We stretch every day to maintain the function of [my child]'s legs after the operation. It doesn't hurt as much now, but it's difficult to get [my child] to understand that it must be done. Getting [my child] to walk with the leg braces has also been a big challenge. But if we stretch every day, we have agreed on a period without braces. I have also become a coach for [my child]'s football team to ensure [my child] can participate in the training sessions. When [my child] gets tired, I can, for example, put [my child] in goal as a keeper.”

(Father, Family 1)

All families described that their active contributions to their child's care required resources in the form of time and flexibility. They needed the ability to be able to plan their paid work, allowing for time away from work and the option to work from home. Several parents mentioned having to work reduced hours to, for example, be able to dedicate 1 day a week to deal with necessary care coordination activities and managing other care tasks. The facilitation of care by families was also addressed in conversations during multidisciplinary coordination meetings, including discussions on participation in exercise and rehabilitation periods, as well as the processing of applications for these services, and families' follow‐up roles.

## 4 Discussion

The findings of this study underscore the lengths families go to navigate care for their child. To prepare themselves to engage with services, families become experts on their child's condition by searching for information and establishing a supportive network. These strategies lay the foundation for a proactive role in their child's care, enabling them to create collaborative relationships with service providers, communicate needs, and identify appropriate services. Additionally, families invest considerable time in effectively managing their child's day‐to‐day care by coordinating services and facilitating and performing care.

### 4.1 Contributions to Family‐Centred Care

By establishing collaborative relationships with providers as a foundation for their child's long‐term care, families create vital family‐provider partnerships that are central to family‐centred services [52]. These partnerships foster open and trusting dialogue, which is crucial for addressing care needs and agreeing on follow‐up approaches. As our study shows, families' needs change over time and are influenced by emerging challenges and contextual factors. Research indicates that the services' ability to adapt to these changes is enhanced by strong relationships between patients, family members and providers, where a partnership approach supports timely and targeted follow‐up care [53, 54, 55, 56].

Families' investment in relationships demonstrates their understanding that quality care encompasses both objective and subjective dimensions [57]. The objective dimension relates to the technical quality of the services, while the subjective dimension pertains to interactions with providers. Our study highlights the importance of the subjective dimensions of care to families, aligning with existing research [57]. For instance, families reported that providers often strive to meet their care needs despite organizational limitations affecting technical quality of care. In situations where their needs were unmet, families maintained collaborative relationships by distinguishing between the technical quality of services and the providers' intentions. This approach allowed families to preserve respectful relationships while advocating for necessary adjustments to their child's care. In line with the core concept of family‐centred care, collaboration based on mutual respect is fundamental [58].

A key concept illustrating the importance of collaborative relationships in achieving high‐quality services is relational coordination [55, 56, 59]. This involves coordinating services through frequent, high‐quality communication supported by shared goals, knowledge and mutual respect. Families contribute to relational coordination through their communication with providers, employing open and direct dialogue with the aim of aligning responsibilities to reach shared goals. These strategies facilitate collaborative goal setting, a critical component of family‐centred care partnerships [60].

Family‐centred care should be coordinated and integrated, addressing the families' services from a long‐term perspective [3, 61, 62]. However, our study shows that achieving service coordination and integration requires families to continually facilitate care provision according to their child's emerging needs. This includes sharing information among all participants, correcting information, addressing interdependencies in care, requesting continuity with service providers and facilitating collaboration between all service providers involved in their care. Parents' roles in coordinating services are well‐documented [17], highlighting the need for improved continuity and coordination in services for children with CP [63]. Like the tasks undertaken by families in this study, research in other settings shows that families often fill knowledge and information gaps between providers, settings and systems [53]. Thus, families possess critical knowledge that is vital for connecting services to holistic approaches through actions that “scaffold” the healthcare system, positively influencing the quality of services [64]. Families make vital contributions to the management of day‐to‐day care by engaging in follow‐up treatment and exercise, providing information during transitions between services, advocating for their child, providing motivation and acting as mediators to help their child to understand the care offered [65, 66]. In doing so, families become co‐creators of resilient healthcare [53, 67, 68], assisting professionals in providing safe and high‐quality care [69].

### 4.2 Supporting and Involving Families

Considering families' important contributions to the co‐creation of family‐centred services, it is crucial to support them in developing and maintaining effective strategies to navigate care. This remains an area in need of improvement, as research shows that providers frequently overestimate their contributions while undervaluing the role parents play in enhancing the quality of care provided to their child [21, 57].

Families' development of effective strategies to navigate care is influenced by their self‐efficacy, various social determinants, as well as the support provided to them [70, 71]. For instance, parents' health literacy has been found to influence their utilization of care services [72]. This study shows that families' strategies, along with their self‐efficacy, develop over time. For example, parents continuously searched for information before and between consultations and discussed the information amongst themselves and with their supportive network. Additionally, families were more confident in applying effective strategies as they gained more parenting experience, particularly when they had multiple children.

Families included in this study were recruited based on their own desire to participate. They had developed effective strategies to navigate care and had important views on how services should meet their care needs. As such, they can be viewed as resilient caregivers, capable of adapting to the physical and psychological requirements of their role [73], by gathering information, mobilizing resources, developing collaborative relationships with providers and establishing supportive social networks [74]. Additionally, the families developed family resilience, which research describes as collectively utilizing internal and external resources to cope with the stressors experienced in long‐term care [75].

Research has shown that the provision of family‐centred care supports families' self‐efficiency [76], leading to numerous positive outcomes improving service quality [77]. However, achieving the goals of family‐centred care necessitates acknowledging families' preferences, as families value different aspects of family‐centeredness, which are influenced by their experience, needs, resources and desire for involvement [78]. As shown in this study, families' preferences are shifting according to challenges they face and their available resources at the time [78]. As such, a situational approach is emphasized, where family‐centred care is achieved by taking the action that is most appropriate for the given situation [79]. Therefore, supporting families in the development of their strategies to navigate care necessitates addressing their preferences and possibilities for involvement, as well as their context and resources, to ultimately give them the support they need. This is particularly important as parents can find the care burden related to their child's services overwhelming [6, 80]. Due to their extensive role in managing their child's care, parents may face time constraints that impact their ability to care for other children, maintain their work schedules, socialize and sustain parental relationships. Over time, an excessive care burden can also affect parents' mental health [12]. This underscores the importance of providing families with adequate support and adopting a dynamic approach to meet their evolving needs as they encounter new experiences and challenges. In doing so parents' resilience capacities are supported [10, 81].

### 4.3 Strengths and Limitations

A strength of this study was the combination of methods used, including interviews and observations, which enabled the collection of information from several sources. Information obtained in interviews was, in several situations, observed during the meetings. Additionally, the families' participation in longitudinal interviews provided insights into the progression of their long‐term care and allowed for the follow‐up of interview topics over time. A limitation of the study is the small sample size. The inclusion of more families and children representing a wider age range could have led to different findings. Another limitation is that the participating families are likely to be health literate and have a desire to participate in research. Their accounts may not represent the navigation experiences of other families. Future research should include a larger sample of families, including children of different age groups and families with diverse backgrounds and experiences.

## 5 Conclusion

To navigate the necessary care services for their child with CP, families applied strategies to (1) become experts on their child's diagnosis, challenges, care needs, and available services; (2) act as proactive participants in their child's care; and (3) manage day‐to‐day care. These strategies were crucial for advocating for their child, ensuring a holistic approach to services through information exchange and coordination between care units and providers, and to daily follow‐up care.

Applying these strategies contributed to family‐centred services with an integrated and holistic approach. This underscores the vital role of families in fully implementing the values of family‐centred care. Identifying families' support needs and their desire for involvement in their child's care can foster the development and maintenance of effective strategies for follow‐up care, allowing for earlier and more effective interventions that meet families' needs, ultimately leading to improved outcomes.

To support families, service providers need to adopt a dynamic approach where follow‐up services are tailored to the families' situations and contexts. Additionally, incorporating families' perspectives in the design of services is essential.

## Author Contributions

**Silje Askeland:** writing – original draft, conceptualization, methodology, formal analysis, investigation, writing – review and editing, project administration. **Veslemøy Guise:** supervision, writing – review and editing, conceptualization, methodology, investigation. **Karina Aase:** supervision, writing – review and editing, conceptualization, investigation, methodology. **Maren Kristine Raknes Sogstad:** writing – review and editing, supervision, conceptualization, investigation, methodology.

## Conflicts of Interest

The authors declare no conflicts of interest.

## Data Availability

The datasets are not publicly available due to confidentiality. However, they can be made available from the corresponding author upon reasonable request and pending ethics clearance from the Regional Committee for Medical and Health Research Ethics in Norway and the Norwegian Agency for Shared Services in Education and Research.
